# Understanding COVID-19 Pathogenesis: A Drug-Repurposing Effort to Disrupt Nsp-1 Binding to Export Machinery Receptor Complex

**DOI:** 10.3390/pathogens10121634

**Published:** 2021-12-17

**Authors:** Sona Vasudevan, James N. Baraniuk

**Affiliations:** 1 Department of Biochemistry, Molecular and Cellular Biology, Georgetown University Medical Center, 3900 Reservoir Road NW, Washington, DC 20057, USA; 2 Division of Rheumatology, Immunology and Allergy, Department of Medicine, Georgetown University Medical Center, 3900 Reservoir Road NW, Washington, DC 20007, USA; baraniuj@georgetown.edu

**Keywords:** COVID-19, Nsp1, nonstructural protein 1, NXF1, NXT1, nuclear export, drug antagonists, structural modeling, structural docking

## Abstract

Non-structural protein 1 (Nsp1) is a virulence factor found in all beta coronaviruses (b-CoVs). Recent studies have shown that Nsp1 of SARS-CoV-2 virus interacts with the nuclear export receptor complex, which includes nuclear RNA export factor 1 (NXF1) and nuclear transport factor 2-like export factor 1 (NXT1). The NXF1–NXT1 complex plays a crucial role in the transport of host messenger RNA (mRNA). Nsp1 interferes with the proper binding of NXF1 to mRNA export adaptors and its docking to the nuclear pore complex. We propose that drugs targeting the binding surface between Nsp1 and NXF1–NXT1 may be a useful strategy to restore host antiviral gene expression. Exploring this strategy forms the main goals of this paper. Crystal structures of Nsp1 and the heterodimer of NXF1–NXT1 have been determined. We modeled the docking of Nsp1 to the NXF1–NXT1 complex, and discovered repurposed drugs that may interfere with this binding. To our knowledge, this is the first attempt at drug-repurposing of this complex. We used structural analysis to screen 1993 FDA-approved drugs for docking to the NXF1–NXT1 complex. The top hit was ganirelix, with a docking score of −14.49. Ganirelix competitively antagonizes the gonadotropin releasing hormone receptor (GNRHR) on pituitary gonadotrophs, and induces rapid, reversible suppression of gonadotropin secretion. The conformations of Nsp1 and GNRHR make it unlikely that they interact with each other. Additional drug leads were inferred from the structural analysis of this complex, which are discussed in the paper. These drugs offer several options for therapeutically blocking Nsp1 binding to NFX1–NXT1, which may normalize nuclear export in COVID-19 infection.

## 1. Introduction

Almost 2 years into the pandemic, we have lost more than 750,000 lives in the United States alone to coronavirus disease 2019 (COVID-19). Though we have several highly efficacious vaccines, we are still far from understanding the full pathogenesis of the disease [1]. Drugs for SARS-CoV-2 have been predicted from protein–protein interaction networks of COVID and host proteins [2], in vitro culture, and drug repurposing strategies [3]. However, none of the efforts have yielded effective human therapies. Instead, they have selected ivermectin, chloroquine, and others that are not effective in clinical trials [4]. We took a fresh approach by assessing SARS-CoV-2 Nsp1 domain and translational suppression of the nuclear RNA export factor 1 (NXF1, Gene ID: 10482) and nuclear transport factor 2-like export factor 1 (NXT1, Gene ID: 29107) proteins [5,6,7].

Nsp1 interferes with several steps in host translation to promote covid viral replication [8]. Nsp1 is the first protein encoded by gene 1 of α- and β-CoVs. It has an N-terminal globular domain and long disordered chain leading to the C-terminal domain. Nsp1 causes translational shutdown of host mRNA by binding to helix 18 (h18) of rRNA in the mRNA entry canal adjacent to the tRNA acceptor site of the 40S ribosome [7]. It interacts with 37 nucleotides of h18 in a fashion like SERBP1 and STM1 [7,9,10]. The C-terminal domain forms a helix KH motif (K164-H165) [7]. The negatively charged patch on the initial short helix (amino acids 154160) binds to a positively charged region of ribosomal protein uS3. The K164–H165 turn is hydrophobic, and binds to a hydrophobic pocket of uS5 [11]. The larger helix (amino acids 166–179) has a positively charged patch that binds to the phosphate backbone of ribosomal h18, as well as to uS5. K164–H165 is essential for inhibition of translation in both SARS-CoV-1 and SARS-CoV-2 [7,12]. Mutation studies that replace K164–H165 with alanine–alanine show that lysine 164 (K164) is essential for Nsp1 to induce cell cycle arrest [13], block IRF3 phosphorylation, and to deplete Tyk2 and STAT2 [14]. These effects prevent induction and expression of antiviral type I IFN. The KH residues have additional functions as they interact with Phe-Gly (FG) motifs of nucleoporins, and are essential for disruption of NUP93 in nuclear pores [5,6,7,8,9,10,12,13,14,15].

Binding of the C-terminal domain to rRNA is sufficient to displace host mRNA, which is followed by endonucleolytic cleavage of nonviral mRNAs [16]. Nsp1 cleavage occurs at the 43S preinitiation complex stage when Nsp1-bound 40S subunits bind to the 5′ cap-proximal region of mRNAs. Cleavage is in the first 50 nucleotides of the host mRNA [8]. In contrast, viral ssRNAs are resistant to Nsp1-induced RNA cleavage because viral ssRNA have 5′ stem loop structures that overcome the translational blockade [16]. Stem loop RNAs are preferentially bound to arginine 124 (R124) of Nsp1, and wind around the globular domain in an electronegative groove [17]. When the C-terminal is latched to the 40S ribosome, the tethered globular domain with its stem loop ssRNA cargo is well placed for delivery to the mRNA acceptor site to begin translation [16,17]. Deletion of amino acids 141–143 (KSF) in the disordered loop of the C-terminal region has been detected by epidemiological genomic sequencing, but is of uncertain pathogenic relevance [16,18].

The multifunctional Nsp1 protein also disrupts the nuclear mRNA export machinery to inhibit host gene expression by binding directly to the complex of NXF1 and NXT1 [5]. NXF1–NXT1 is the principal factor that mediates docking and translocation of messenger ribonucleoprotein complexes through the nuclear pore complex (NPC) from nucleus to cytoplasm [8,19,20]. NXF1–NXT1 binds the transcription-and-export complex (TREX) that includes THO, UAP56, DDX39B, ALYREF (Aly/REF export factor), and proteins that bind to 5’ 7-methylguanosine caps, spliced intron sites, and polyadenine tails of mRNAs [21]. It is not known if Nsp1 can interfere with these nuclear export proteins. The exact binding interactions are still under investigation [19,20,21,22]. Nsp1 did not impair the RNA-binding ability of NXF1 [5].

NXF1–NXT1 also participates in a parallel export system, the chromosome region maintenance 1 (CRM1, XPO1 exportin 1) export mechanism, that conveys complexes containing eukaryotic initiation factor 4E (EIF4E), a 5’ cap chaperone protein. These complexes include cytokine and growth factor mRNAs that contain 5’ caps and the ~50 nucleotide eIF4E sensitivity element (4ESE) in their 3’ UTR [23]. This population of mRNAs may include interferon mRNAs that must be translated for effective early antiviral defense, but that are inhibited by Nsp1 during COVID infection. Ribosome subunits (GO:0000054 biological process ribosomal subunit export from nucleus) and RNAs with complex secondary structures, such as stem loops (constitutive transport elements, CTE) [24], are also transported by NXF1–NXT1 complexes. Nsp1 is a cytoplasmic protein without a nuclear localization signal, but the interaction with NXF1–NXT1 or nucleoporin binding by its C-terminal helices may explain its association with the nuclear pore [23,24,25].

NXF1 interacts with phenylalanine-glycine (FG) motifs on nucleoporin proteins in the nuclear pore complex to facilitate transport. Nsp1 can interrupt interactions between NXF1 and nucleoporin proteins, including Nup358, Nup214, and Nup98. Overexpression of COVID-19 Nsp1 in HEK cells disrupted Nup93 localization around the nuclear envelope and the nuclear-cytoplasmic distribution of nucleolin, but did not disrupt the nuclear lamina, or trigger proteolytic degradation [15]. Binding of Nsp1 to NXF1–NXT1 prevents docking of the cargo mRNA complex to the nuclear pore, and impairs host mRNA translocation to the cytoplasm, and subsequent translation [15]. Alhough there have been efforts to identify drugs to the Nsp1 protein domain [26,27], none disrupt its interactions with NXF1–NXT1.

These findings led us to explore the structural interactions between Nsp1 and the NXF1–NXT1 complex, and to identify drug candidates that could disrupt the interaction of Nsp1 with NXF1–NXT1.

## 2. Methods

Protein Data Bank (rcsb.org (accessed on 1 August 2021)): 3D-protein structures used in this manuscript were retrieved from the Protein Data Bank structure of the LRR and NTF2-like domains of NXF1 complexed with NXT1 (PDB-ID 4WYK), and crystal structures of NSP1 from SARS-CoV-2 (PDB-ID: 7K3N) were used in this study. PDB is the single archive of structural data of biological macromolecules worldwide [28]. PDB is a repository that includes experimentally determined structures using the methodologies of X-ray crystallography, nuclear magnetic resonance spectroscopy, and electron microscopy. All the structures used in the manuscript are experimentally determined by X-ray crystallography.

Protein–Protein Docking: Docking of Nsp1 (PDB-ID: 7K3N) to the structure of the LRR and NTF2-like domains of NXF1 complexed with NXT1 (PDB-ID: 4WYK, and PBD 1JN5) was carried out using the protein–protein docking software, ClusPro [29]. ClusPro software is run on a server that can be assessed at https://cluspro.org/login.php (accessed on 10 September 2021). It provides the flexibility of using six different energy functions depending on the type of protein. Default parameters were used for the docking. As for input, the server requires two PDB files: one for each protein that is to be docked. The server provides the top 10 energetically favorable docked structures. The structure that had the least favorably energy and fulfilled geometrical constraints was used for further analysis.

Docking of FDA-Approved Drugs Using Molecular Operating Environment (MOE) Software: 1993 FDA-approved drugs were obtained from the e-drug database for docking analysis in SDF format [30]. These drugs were docked to the structure of the LRR and NTF2-like domains of NXF1 complexed with NXT1 (PDB-ID: 4WYK) using the Molecular Operating Environment (MOE) software [31]. Three-dimensional protonation and energy minimization were done with the compute option within MOE until a gradient of 0.05 was reached. Polar hydrogens were added, and the site finder feature was used to search for binding pockets. Standard parameters were used for docking. The top hit from the site-finder coincided with the Nsp1 binding region. The docked compounds were ranked based on their docking scores (S). The compounds with the best docking scores were further evaluated for their molecular interactions with the proteins.

PDBSum [32]: PDBsum is a freely available web server (www.ebi.ac.uk/pdbsum/ (accessed on 1 August 2021)) providing structural information on the entries in the Protein Data Bank (PDB). The analyses are primarily image-based, and include protein secondary structure, protein–ligand, protein–protein, protein–DNA interactions, and many others. The generate feature within the PDBSum was used for obtaining the binding interaction residues in the complex between NXF1–NXT1 and Nsp1.

PyMOL (www.Pymol.org (accessed on 1 August 2021))**:** PyMOL is a visualization software used to create all the figures in this manuscript.

COVID Gene List (Table 1): The set of genes implicated in COVID were mined from the literature by direct searches using Pubmed (https://www.ncbi.nlm.nih.gov/pubmed (accessed on 1 July 2020–11 January 2021)) and LitCovid (https://www.ncbi.nlm.nih.gov/research/coronavirus/ (accessed on date 1 August 2021)). Genes responsible for critical, severe, and mild forms of the disease were retrieved from the OpenTargets database. These searches resulted in a total of 31 genes that formed our gene set. The in-depth analysis of these genes is beyond the scope of this manuscript, and will be described elsewhere. We have, however, added the functional classifications to Table 1.

## 3. Results and Discussions

Based on the ability of SARS-CoV-2 Nsp1 to inhibit mRNA export [5], we hypothesized that a 3D model would shed light on the mode of action of Nsp1, and its ability to block the interaction of NXF1 with the nuclear pore complex. Currently, there are no structures available for NXF1–NXT1–Nsp1 complex. The sequences of Nsp1 (PDB-ID: 7K3N) [33] and NXF1–NXT1 (PDB-ID: 4WYK [24]; PBD-ID 1JN5 [34]) have been truncated to facilitate the original structural modeling.

### 3.1. NXF1–NXT1

**Figure 1:**
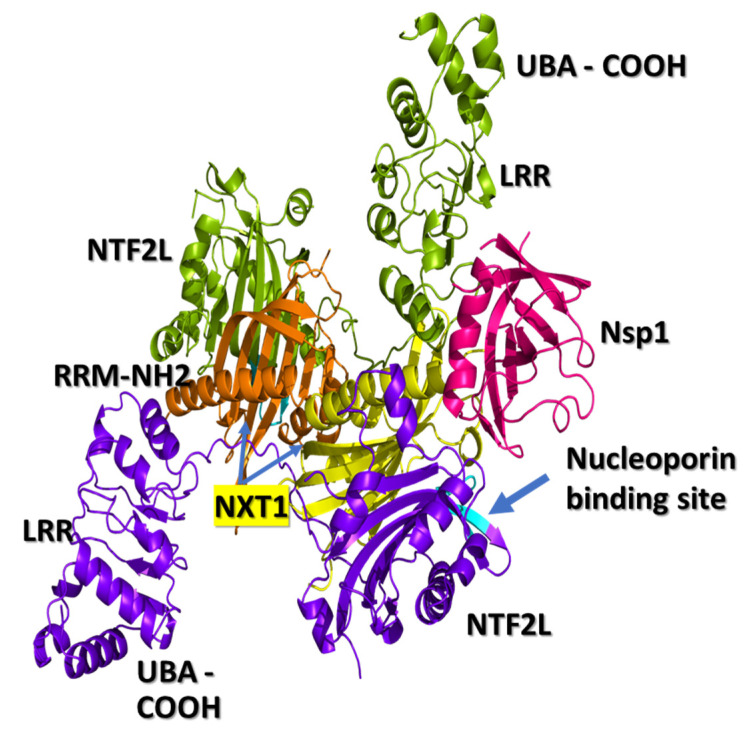
NXF1–NXT1 and Nsp1 complex. Interactions between Nsp1 and the NXF1–NXT1 complex were found using truncated NXF1 (PDB-ID: 4WYK) (19). NXF1 has 5 domains extending from the N-terminal RRM, LRR, loop, NFT2L, and C-terminal UBA domains, but only the truncated complex of LRR, NFT2L, and NXT1 was visualized. RRM is folded to the side of LRR, and contributes to the mRNA binding surface. UBA is folded under NFT2L for nucleoporin binding. Two NXT1 proteins (yellow and orange) were bound to two NXF1 proteins (green, upper; blue, lower) to form a “pancake”. One surface is for RNA binding. Nsp1 (magenta) interacts with NXT1 and the LRR, and NTF2L domains of NXF1 on the “side” of the “pancake”. The complex was rotated to show the nucleoporin binding site of NXF1 (cyan). Figure generated using PyMOL.

The three-dimensional structure of the NXF1–NXT1 complex (PDB-ID: 4WYK) [24] approximates a Z-shaped “pancake” (Figure 1). NXF1 contains an N-terminal RNA recognition motif (RRM), a leucine-rich repeat domain (LRR), a nuclear transport factor 2-like domain (NTF2L), and a ubiquitin-associated domain (UBA). The small NXT1 (nuclear transport factor-like export factor 1) protein interacts with the NTF2L domain of NXF1. Two of these heterodimers join based on contacts between the NXT1 domains, then are locked into position by a hydrophobic loop from LRR to NTF2L [24,34]. One side of the pancake is the RNA binding surface, and the flip side binds nucleoporins.

The N-terminal RRM domain curls back onto LRR to form a flat RNA binding module on one side of the pancake. The RRM–LRR of NTF2L surface can interact with phosphate backbones of ssRNA sequences. There may be some element of secondary structural specificity, as nucleotides of constitutive transport element (CTE) stem-loop structures of some retroviral RNAs interact with RRM residues 123, 125, 126, 128, 151–154, 156, 158, 190, and 192; LRR residues 233, 248, 276, 278, 279, 304, 305, and 307; and NTF2L residues 429–469. Such constraints may also apply to preferential binding to 5′ capped, intron splice sites and 3′ polyA sequences. Contact sites for interactions with ALYREF and other nuclear export proteins have not been structurally mapped [22].

The opposite surface of the NXF1–NXT1 pancake exposes nucleoporin binding regions in NTF2L between positions Q486-L491, A519-P521 and L527 (Figure 1) that bind tight phenylalanine-glycine (FG) turn motifs and the C-terminal UBA domain that binds to FXFG sequences of nucleoporins [20]. FG residues from NUP42, NUP214, and NUP98 project into the lumen of the nuclear pore, and lock onto P521 (proline 521) in a hydrophobic pocket of NTF2L (PBD 1JN5) [34]. The phenylalanine stacks against P521 in the pocket (PDB 1JN5). Glycine is also coordinated with P521. The complex is surrounded by L383, L386, Q486, L491, A519, and L527. The interaction is relatively weak, which enables rapid exchange between the RNA transport complex and nucleoporins, allowing ribonucleoprotein translocation through the pore into the cytoplasm. Entry of the complex into the nuclear pore is facilitated by DEAD box ATPase UAP56 at the nuclear margin, followed by cytoplasmic dissociation by DDX19. Unlike NFT2, the NFT2L domain of NXF1 may not be able to bind RanGDP proteins that assist with RNA dissociation on the cytoplasmic face of the nuclear pore, and which were found in the COVID-related protein lists (Table 1*).*

### 3.2. Nsp1 Docked to NXF1–NXT1

**Figure 2:**
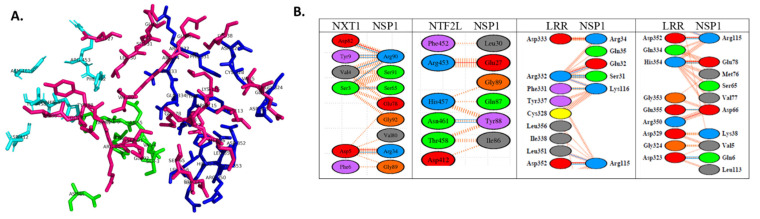
Nsp1 binding pocket and amino acid interactions. (**A**) Stick representation of the binding pocket shows Nsp1 residues (magenta) that interacted with NXT1 (cyan), and the NTF2L (green) and LRR (dark blue) domains of NXF1. Individual residues are labeled. Figure generated using PyMOL. (**B**) Interacting amino acids from each protein were color-coded for acidic (red), basic (blue), hydroxy (green), uncharged (orange), aliphatic (grey), and aromatic (purple) residues. Lines indicate salt-bridges (blue), hydrogen bonds (heavy red cross hatching), and other interactions (dotted red). Figure generated using PDBSum, and Nsp1 (PDB-ID: 7K3N) and NXF1–NXT1 (PDB-ID:4WYK).

We docked the structure of Nsp1 (PDB-ID: 7K3N) to the NXF1–NXT1 (PDB-ID:4WYK) complex using the ClusPro server [33]. Nsp 1 binds to a surface on the “edge” of the “pancake” that is formed by the LRR and NTF2L domains, and the N-terminal of NXT1 (Figure 1). Nsp1 utilizes 27 residues to interact with 29 residues from the complex. Binding was stabilized by 20 hydrogen bonds, 8 salt-bridges, and 224 non-bonded interactions (Figure 2). The involvement of salt-bridges indicates strong interactions with Nsp1.

**Figure 3:**
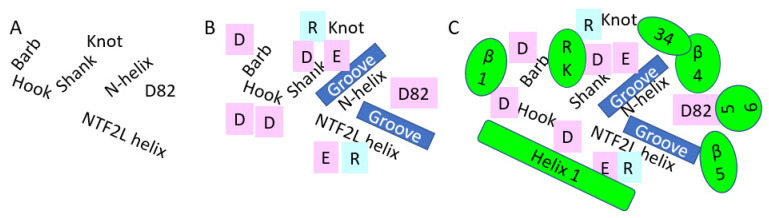
Presumptive Nsp1 binding surface on NXF1–NXT1 domains. Nsp1 bound to the side of the “pancake” where the LRR, NXT1, and NFT2L domains meet. (**A**) LRR was folded into a “hook” with a knot-alpha helical shank-loop (hook) and barb. The shank was roughly parallel to the N-terminal helix of NXT1. D82 of NXT1 was adjacent to its helix. The lateral helix of NFT2L completed the binding surface. (**B**) These structures contributed charged acid (pink) and basic (blue) amino acid residues to the surface. Spaces between them formed hydrophobic grooves. (**C**) Nsp1 domains (green) were superimposed on this hydrostatic landscape. The start of beta 1 interacted with the barb. Alpha helix 1 bridged the hook, the end of the NXT1 helix and NFT2L helix. The loop to helix 4 wrapped around the knot, leading to the interaction of helix 4 with the NXT1 helix. The antiparallel beta 5 lay over the groove beside the NFT2L helix, then formed a loop over D82. R124K125 hoovered over the charged LRR knot and shank. We propose that drugs with cationic groups could interfere with the charged interactions, whereas hydrophobic segments may occupy the grooves.

**Figure 4:**
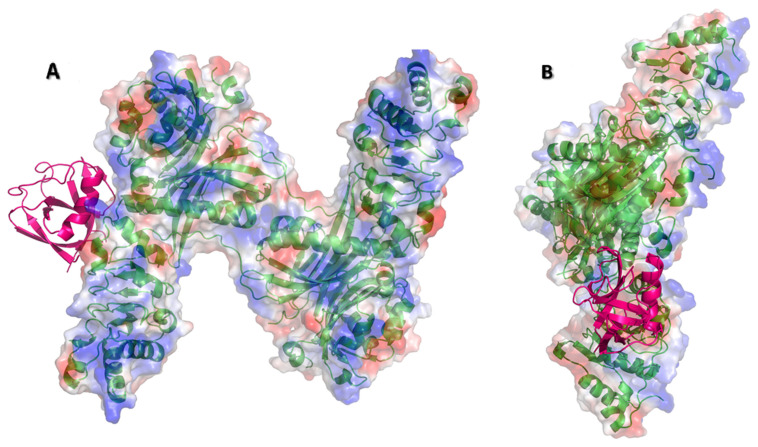
Electrostatic binding surfaces. (**A**) Nsp1 (PDB-ID: 7K3N, magenta) was docked to NXF1–NXT1 (PDB-ID:4WYK) using the ClusPro server. The N-terminal RRM and C-terminal UBA domains of NXF1, and N-terminal and C-terminal domains of Nsp1 were truncated to provide stable structures for the original crystallography studies. Electrostatic representations (acidic red to blue basic) of the NXF1–NXT1 complex (green backbone) indicate strongly charged residues in the Nsp1 binding region and RNA binding surface. (**B**) The complex was rotated 90°, showing the RNA binding surface (blue, basic) to the right.

The interacting face of NXF1–NXT1 was formed by the LRR “hook” with a knot at its top extending to the alpha helical shank, loop of the hook, and helical barb (Figure 3). Adjacent was the N-terminal helix of NXT1, then a loop exposing NXT1 aspartate 82 (D82). A helix of NTF2L was the fourth contributing structure. The knot and hook of LLR, nexus of LLR–NTF2L–NXT1 domains, and D82 of NXT1 were charged, whereas the grooves between the shank and the NXT1 alpha helix and NTF2L helix were relatively hydrophobic. Charged moieties on the domains facilitated interactions (Figure 4).

Based on the interacting amino acids (Figure 2), the proximal end of beta 1 from Nsp1 opposed the barb. Alpha helix 1 abutted the NTF2L helix, and continued along the loop and helix of the LRR hook (Figure 3). The loop between beta 3 and beta 4 interacted with the knot at the upper end of the LRR hook proximal to loop 3. Beta 4 lay over the N-terminal helix of NXT1, and abutted the adjacent LRR knot. Beta 5 lay over the NTF2L helix, whereas the contiguous loop bonded D82 and the N-terminal helix of NXT1. K124R125, which follows beta 7, interacted with charged residues in the knot and shank of LRR. Other beta sheets and loops of Nsp1 were directed away from the NXF1–NXT1 complex, and were unlikely to interfere with the nucleoporin FG loop binding by P521 of NTF2L. However, Nsp1 reduces binding of NXF1 to mRNA export adaptors, such as ALY/REF and UAP56, but not THOC6 [5]. Future structural modeling will be required to determine how Nsp1 interferes with these and other nuclear export proteins.

The binding of Nsp1 to NXF1–NXT1 may provide an additional virulence mechanism. NXF1–NXT1 can bind to stem loops that occur on viral ssRNA [35]. If NXF1–NXT1 become stranded in the cytoplasm, the complex may bind to stem loop COVID ssRNA [17]. Capture of the NXF1–NXT1–ssRNA by Nsp1 would target the complex to the 40S ribosome where the long C-terminal motif would anchor the complex to the acceptor site, and preferentially enhance translation of viral proteins [35].

### 3.3. Molecular Docking of Drugs to Nsp1 Binding Site on NXF1–NXT1

The modeling offered several possibilities for Nsp1 to hijack the translational machinery. This opened the door to assess drug re-purposing to interfere with Nsp1 pathology. We used the e-Drug3D database, which contained 1993 FDA-approved drugs to dock to the structure of the NXF1–NXT1–Nsp1 complex to disrupt the interaction of Nsp1. Two dockings were performed. The first had all drugs, including peptides, but the second was limited to small molecular weight compounds (<500 Da) with fewer than eight rotatable bonds. The results of the dockings are provided in Table 2 and Table 3.

The top hit of all dockings was ganirelix, with a docking score of −14.49 (Table 2). Ganirelix competitively antagonizes the gonadotropin-releasing hormone receptor (GNRHR) on pituitary gonadotrophs, and induces rapid, reversible suppression of gonadotropin secretion. SARS-CoV-2 infection has been associated with altered gonadotropin, androgen, and testosterone secretion that may disrupt male reproduction and fertility, and female menstrual cycles. The conformations of Nsp1 and GNRHR make it unlikely that they interact with each other [36,37].

**Figure 5:**
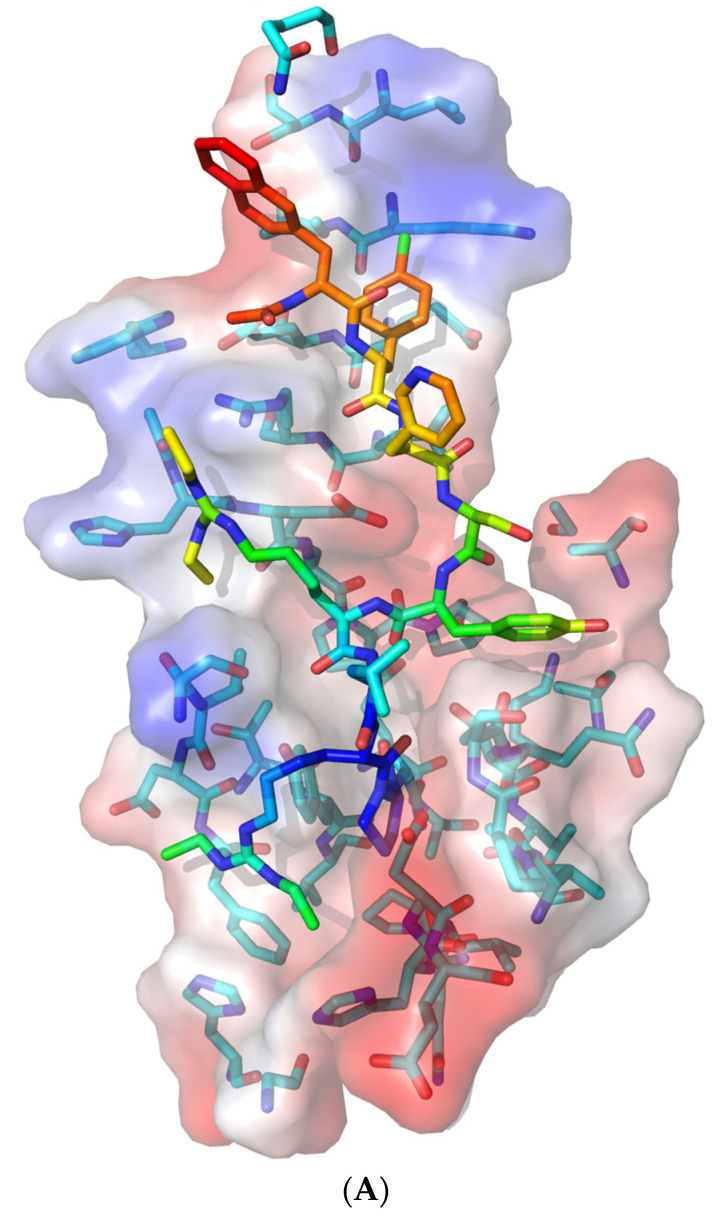

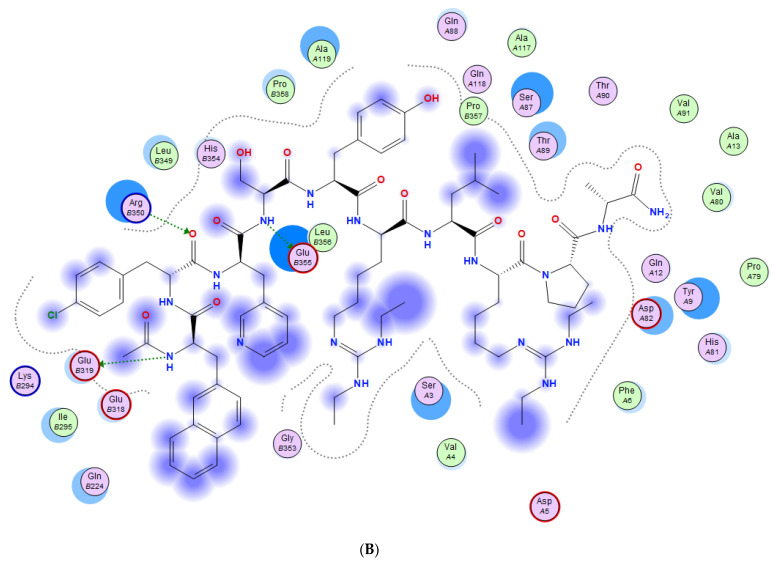
Ganirelix binding pocket on Nsp1. (**A**) Ganirelix is shown as rainbow sticks on the acidic (red) and basic (blue) electrostatic surface of Nsp1. The Nsp1 peptide backbone is faded for contrast. The majority of ganirelix residues interact with the NXF1–NXT1 binding surface of Nsp1, which predicts that ganirelix will inhibit binding of Nsp1 to the complex. (**B**) Nsp1 amino acids that are hydrophobic (green circles) and polar (pink circles) form the binding pocket for exposed ganirelix cationic groups (blue). The diagrams are oriented to show the aromatic N-terminal amino acids at the top, homoarginine residues in the mid portion, and C-terminal tail at the bottom.

Ganirelix has a hydrophobic N-terminal with aromatic substituted amino acids, two cationic diethylhomoarginine groups in the midsection, and C-terminal Pro-Ala (Ac-D-2Nal-D-Phe(4-Cl)-D-3Pal-Ser-Tyr-D-hArg(Et,Et)-Leu-hArg(Et,Et)-Pro-D-Ala-NH2, PubChem CID 16130957). Ganirelix was unique by virtue of the diethylhomoarginine moieties, and was about one order of magnitude more potent for disruption of Nsp1 from NXF1–NXT1 than the other drugs. However, the large flat cationic sidechains could intervene between the acidic LRR knot and shank, NTF2L helix, or NXT1 D82, as well as the charged Nsp1 alpha helix and C-terminal R124K125 residues (Figure 3). The ketones of the amide groups offer opportunities for peptide bond backbone interactions (see below). Ganirelix forms a tight fit in the electrostatic binding pocket of Nsp1 (Figure 5).

Our search did not identify any small nonpeptide GNRHR antagonists. These drugs have very different chemical structures honed to bind deep in the central cavity of the 7 transmembrane receptor structure (PDB 7BR3) [36,37]. Therefore, it is unlikely that activity against GNRHR, per se, will be important for determining the conformation of ganirelix for blocking Nsp1.

The peptide drug list was inspected for cationic and aromatic sidechains (Table 2). Other GNRHR antagonists featured arginine instead of diethylhomoarginine, and had aromatic amino acids: triptorelin, cetrorelix, leuprolide, and nafarelin. Icatibant, a bradykinin B2 receptor antagonist peptide, had three arginine cations plus aromatic sidechains. Afamelanotide is a 13 amino acid peptide analog of alpha melanostatin-stimulating hormone with one arginine plus aromatic amino acids. Degarelix and abarelix were GNRHR antagonist peptides with aromatic amino acids, but lacked arginine. Ceruletide and sincalide are peptide cholecystokinin agonists that lacked arginine. Etelcalcetide is a calcium sensing receptor agonist peptide with four arginines, but lacked aromatic amino acids. Ranking these characteristics by binding coefficient (S) suggested that drugs with a decapeptide backbone, cationic group (preferably diethylhomoarginine), and aromatic sidechains would have greater efficacy for blocking Nsp1.

An alternative mechanism was offered by sulfated cyclic compounds (Table 2). Fondaparinux was a sulfated glucopyranoside that neutralizes activated factor X (Factor Xa) to prevent coagulation. Colistimethate is a sulfated cyclic peptide that acts as a broad spectrum polymyxin antibiotic. Less potent was sugammadex, an octosaccharide cyclodextrin used to reverse neuromuscular blockade, which contained substituted thiopropionic acid groups, but lacked an amino cationic structure. These cyclic compounds offered many hydroxyls, ketone, and carboxyl groups that may have disrupted the peptide bond and charged sidechain interactions between Nsp1 and NXF1–NXT1.

**Figure 6:**
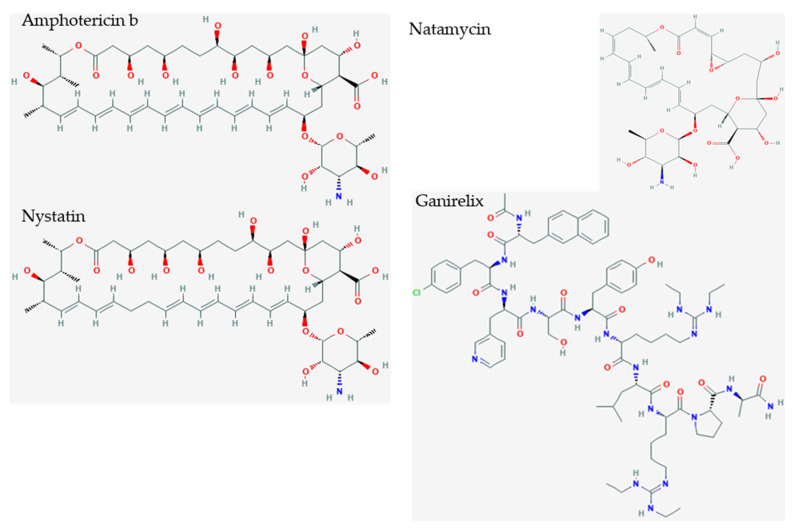
Small polyene drug candidates and ganirelix. Amphotericin b (S = −10.10), nystatin (S = −9.46), and natamycin (S = −7.74) are polyene amphoteric macrolide antibiotics that bind ergosterol in fungal membranes, and cause membrane depolarization, altered membrane permeability, and membrane pore formation. They have saw tooth alkene polymers along the sawblade, ketones along the spine of the sawblade, and aminoglycoside cation as a handle. Ganirelix (S = −14.49) is more potent and can be morphed to show an aromatic analog to the aliphatic sawblade, backbone of peptide bonds with ketones, and cationic diethylhomoarginine groups.

**Figure 7:**
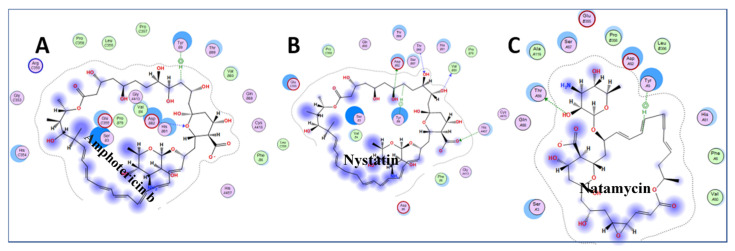
Amphotericin b (**A**), nystatin (**B**) and natamycin (**C**) binding pocket interactions. The ligands are shown in Figure 6. Natamycin supported our conjecture. However, the longer rings of amphotericin and nystatin acted like lassos by fitting to hydrophobic grooves, and encircling amino acids on the binding surface. These structures will need to be confirmed by future crystal structure examinations. The light pink circles are polar amino acids, light pink circle with a red circle around it is acidic amino acids, pink with blue circle is basic amino acids, green circle is hydrophobic at a van der Waals distance, bluish hazy circles indicate ligand exposure, light blue circles indicate NXT1–NXF1 exposure.

Inspection of the small drugs found three antifungal agents: amphotericin b, nystatin, and natamycin (Figure 6) Natamycin, amphotericin, and nystatin have a saw-like shape with alkene teeth, oxygenated (ketone) spine, and amino sugar handle. Natamycin is smaller and likely to retain this shape in contrast to the looser and more mobile compounds with larger rings. The structure suggests a stiff platform with hydrophobic and hydrophilic edges, and a charged appendage that lies at an angle from the plane of the platform. This shape suggests insertion of the alkene saw blade into a hydrophobic groove so that the oxygen atoms on the spine of the saw can interact by van der Waals forces with peptide bonds and other sidechains. The amino glycoside “handle” may interact with the surface of charged molecules. In this scenario, nystatin and amphotericin would be scaled up versions of natamycin. These polyene macrolides bind to ergosterol in the fungal membrane, and cause severe adverse events in humans by binding to cholesterol, especially when concentrated in renal tissues [38]. Natamycin, amphotericin, and nystatin show different modes of binding, and are very different from the interactions with ganirelix. Figure 7 shows the binding pockets of natamycin, amphotericin, and nystatin, as well as their interactions with the NXT1–NXF1 complex.

This scenario was of interest because ganirelix can be flexed into a saw-like shape with aromatic groups on the sawblade, ketones of peptide bonds along the spine of the saw, and diethylhomoarginines as the large flat charged cationic “handles”. The aromatic groups would provide greater selectivity for hydrophobic interactions and π stacking than the alkene edge of amphotericin, nystatin, and natamycin.

**Figure 8:**
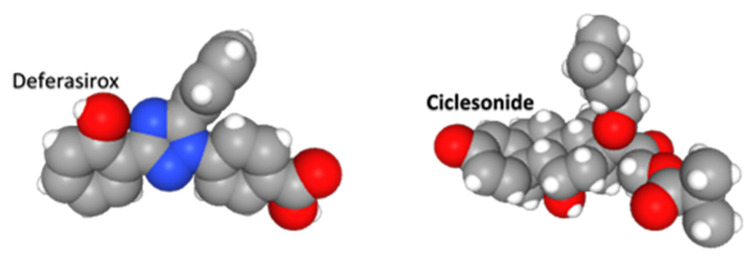
Deferasirox and ciclesonide. Deferasirox is an achiral, tridentate triazole derived from salicylic acid that chelates trivalent (ferric) iron. Ciclesonide is an inhaled glucocorticoid used to treat asthma.

Deferasirox is an achiral, tridentate triazole derived from salicylic acid that chelates trivalent (ferric) iron (Figure 8). It was used as the prototype for a drug configuration with a flat platform substituted with oxygen and nitrogen ring structures, and a covalently bound, but rotatable, group that is constrained orthogonal to the plane of the platform. This is a “boat and sail” configuration. Idarubicin, azelastine, ixabepilone, minocycline, and tadalafil share this general shape. We suggest the flat hydrophobic “boat” and “sail” can be inserted between opposing helices and beta sheets, and intrude into cavities between adjacent loops of the Nsp1 and NXF1–NXT1 complex.

Ciclesonide is an inhaled glucocorticoid used to treat asthma (Figure 8). It is a prodrug that is converted by local respiratory mucosal esterases to the active metabolite desisobutyryl-ciclesonide (des-CIC). This glucocrticoid agonist has anti-inflammatory properties by binding NFKB and AP1, and activating glucocorticoid response elements (GRE). In addition to being selected as a drug for Nsp1–NXF1–NXT1, it can also inhibit SARS-CoV-2 Nsp15 endonuclease by interacting with Tyr344 and adjacent amino acids in a hydrophobic groove [39]. It is not cationic. Clinical trials are required to determine if ciclesonide has any advantage over other inhaled steroids for treatment of pulmonary manifestations in COVID-19.

Our targeted structural docking search proposes that the secondary structures, and cationic and hydrophobic properties of repurposed drugs shared a new niche to intervene between Nsp1 and the NXF1–NXT1 complex. This orientation may be different from presumed mechanisms of action on current FDA-approved clinical targets.

The electrostatic nature of the drugs requires they be investigated as cationic amphiphilic drugs (CAD) that can induce phospholipidosis [40,41]. This nonspecific drug side effect disrupts lipid homeostasis in endosomes, lysosomes, and other membrane-bound organelles [42]. Lipid processing is critical for viral replication, and has become a target for COVID therapies. Phospholipidosis depends on the physicochemical properties of the chemicals, and does not reflect specific target-based drug activities. Other compounds include small molecules that inhibit lysosomal phospholipases and acid sphingomyelinase, cross the blood-brain barrier, and violate Lipinski’s rule of five for hydrogen bonding and drug permeability [40,41,42,43,44].

Cationic amphiphilic drugs were identified as COVID candidate drugs early during hypothesis-free in vitro drug discovery searches that identified agents active against sigma receptors. The list has now been expanded to include chloroquine, amiodarone, haloperidol, clemastine, sertraline, tamoxifen, and many others. However, there is a direct relationship between phospholipidosis and antiviral activity against SARS-CoV-2 in cell cultures [40]. The drugs have cationic amine groups that are hydrophilic at physiological pH (pKa > 7.4), and small rigid aliphatic or aromatic hydrophobic side chains (organic solvent partition coefficients logP > 3.0) [43,44].

Ganirelix, amphotericin, and many of our other candidate drugs (Table 2 and Table 3) meet the criteria of being cationic, hydrophilic, and hydrophobic, based on their arginine sidechains, peptide backbones, and aromatic groups. They may have the capacity to induce phospholipidosis. However, they were discovered by hypothesis-directed structural modeling of the protein structures and may be effective as Nsp1 antagonist drugs at lower doses than those that induce phospholipidosis.

Another strategy to target Nsp1 is a tailored titanium dioxide nanoparticle that binds to E41, Q44, and H45 in the C-terminal end of helix 1 [45].

Structural modeling provides novel insights into Nsp1 pathology and virulence, and predicted candidate drugs. This method relies on crystallized or cryoimages of protein conformations, and assumes these will be the most optimal for predicting other protein–protein and protein–drug docking. It will be essential to confirm that our selected drugs can limit COVID infection in vitro [40] at nanomolar concentrations below the micromolar levels associated with phospholipidosis in dose–response experiments, and be active in animal infection models. New structural studies of the Nsp1–NXF1–NXT1 complex with candidate drugs are needed to verify the preliminary discoveries, and to verify the predicted drug effects. These drugs also need to be validated using in vitro studies. This is our next step, and we are hopeful that these will be validated at some point in the near future.

## Figures and Tables

**Table 1:** Set of genes implicated in COVID-19 gathered from literature searches.

Function	Official Gene Symbol	Protein Names
Extracellular	ACE	Angiotensin-converting enzyme (EC 3.4.15.1)
	ACE2	Angiotensin-converting enzyme 2 (EC 3.4.17.23)
	CRP	C-reactive protein
	DPP4	Dipeptidyl peptidase 4 (EC 3.4.14.5)
	F3	Coagulation factor III
	SERPINE1	Plasminogen activator inhibitor 1 (PAI)
	PKP2	Plakophilin-2
Immune	TNF	Tumor necrosis factor (Cachectin)
	IL6	Interleukin-6
	IFNG	Interferon gamma
	CD4	T-cell surface glycoprotein CD4
Nuclear transport	NTF2	Nuclear transport factor 2
	NXF1	Nuclear RNA export factor 1
	NXT1	NTF2-related export protein 1
	TPR	Nucleoprotein TPR
	XPO1	Exportin-1
	RANBP1	Ran-specific GTPase-activating protein
	RANBP2	RAN binding protein 2 (Nup358 Nuclear pore complex protein)
Nuclear	POLA2	DNA polymerase alpha subunit B
	PRIM1	DNA primase small subunit (DNA primase 49 kDa subunit)
	PRIM2	DNA primase large subunit (DNA primase 58 kDa subunit)
	DDX39B	Spliceosome RNA helicase DEAD box protein
	SARS1	Serine--tRNA ligase, cytoplasmic
Apolipoprotein	APOE	Apolipoprotein E
	APOL1	Apolipoprotein L1
Metabolism	INS	Insulin
	G6PD	Glucose-6-phosphate 1-dehydrogenase
	COLGALT1	Procollagen galactosyltransferase 1 (EC 2.4.1.50)
	LGALS3	Galectin-3 (Gal-3) (35 kDa lectin)
	SLC17A5	Sialin (H(+)/nitrate cotransporter)
	MB	Myoglobin
	AQP4	Aquaporin-4

**Table 2:** e-Drug3D peptide mimetics. 5 Top hits with docking score values −12.0) from 1993 FDA-approved drugs selected for docking to the NXT1-Nsp1 complex with the purpose of disrupting the Nsp1 interaction.

Drug	Chemical Type	Target	Outcome	S
Ganirelix	decapeptide	GnRH antagonist	competitive gonadotropin-releasing hormone antagonist reduces estrogen and testosterone	−14.49
Triptorelin	decapeptide	luteinizing hormone releasing hormone (LHRH) agonist	reversibly represses gonadotropin-releasing hormone secretion to decrease LH and FSH, and estrogen and testosterone	−13.58
Colistimethate	methanesulfonate derivatives of cyclic polypeptides colistin A and B	surfactant that disrupts bacterial cell membrane	broad-spectrum polymyxin antibiotic against most aerobic Gram-negative bacteria	−13.38
Fondaparinux	synthetic glucopyranoside	activates antithrombin III	neutralizes activated factor X (Factor Xa), and prevents thrombin formation	−13.26
Cetrorelix	decapeptide	gonadotrophin-releasing hormone (GnRH) antagonist	blocking GnRH prevents LH secretion, and reduces estrogen and testosterone	−13.23
Leuprolide	nonapeptide	gonadotropin-releasing hormone (GnRH) receptor agonist	inhibits pituitary FSH and LH secretion, causing decline of testosterone and estradiol	−13.04
Icatibant	decapeptide	bradykinin B2 receptor (B2R) antagonist	prevents B2R-mediated vasodilation, vascular permeability, swelling, inflammation, and pain	−12.76
Afamelanotide	peptide	alpha melanocyte-stimulating hormone (α-MSH) analogue	erythropoietic protoporphyria	−12.70
Nafarelin	peptide	gonadotropin-releasing hormone agonist		−12.50
Degarelix	peptide	gonadotrophin-releasing hormone (GNRH) antagonist		−12.06
Sincalide	C-terminal octapeptide of CCK, CCK8	cholecystokinin agonist	induces gallbladder smooth muscle contraction for bile and pancreatic enzyme secretion	−12.04

**Table 3:** Small molecules docking results. Top hits for small molecules with fewer than eight rotatable bonds, and MW < 500 from 1993 FDA-approved drugs selected with e-Drug3D for docking to the NXF1–NXT1–Nsp1 complex to disrupt the Nsp1 interaction. S is the docking score.

Drug	Target	Outcome	S
Amphotericin b	ergosterol binding	membrane pore formation	−10.10
Nystatin	ergosterol binding	membrane pore formation	−9.46
Rifaximin	beta-subunit of the bacterial DNA-dependent RNA polymerase	antibiotic	−8.75
Deferasirox	binds trivalent (ferric) iron	iron chelation	−8.26
Idarubicin	DNA binding	DNA breaks	−7.77
Natamycin	ergosterol binding	membrane pore formation	−7.74
Loratadine	histamine H1R antagonist	“inverse agonist” for H1R antagonism	−7.73
Ciclesonide	glucocorticoid receptor agonist	inhaled asthma drug	−7.68
Clozapine	5-HT2A, 5-HT2C, D1-D4 dopamine receptors	D4R antagonist	−7.64
Praziquantel	Schistosome calcium ion channels	anthelmintic	−7.62
Azelastine	histamine H1R antagonist	antihistamine	−7.58
Ixabepilone	microtubules	cell cycle specific antimicrotubule agent	−7.54
Minocycline	aminoacyl-tRNA binding to bacterial 30S ribosome	inhibit protein synthesis	−7.48
Tadalafil	inhibits phosphodiesterase type 5 (PDE5)	increases cGMP to enhance erectile function	−7.45

## Data Availability

All data used in this study can be obtained from the public domain.
